# Pycnodysostosis Revealed by Recurrent Fractures: Report of Two Cases

**DOI:** 10.7759/cureus.109629

**Published:** 2026-05-25

**Authors:** Abdelkhalek Hamoutahra, Hafid Talha

**Affiliations:** 1 Pediatric Surgery Department, Faculty of Medicine and Pharmacy, Moulay Hassan Ben Mahdi Regional Hospital, Laayoune, MAR; 2 Laboratory of Health Sciences of Errachidia, Faculty of Medicine and Pharmacy of Errachidia, Moulay Ismail University of Meknes, Errachidia, MAR; 3 Pediatric Surgery Department, Moulay Ali Cherif Regional Hospital Center, Errachidia, MAR

**Keywords:** complicated fractures, genetic bone anomalies, orthopedic management, pycnodysostosis, recurrent fractures

## Abstract

Pycnodysostosis is a rare autosomal recessive skeletal dysplasia caused by cathepsin K deficiency and characterized by osteosclerosis and bone fragility. Recurrent low-trauma fractures and delayed healing represent major orthopedic challenges, particularly when medullary canal narrowing limits surgical options. We report the case of two brothers born to consanguineous parents who presented with short stature, characteristic craniofacial dysmorphism, acro-osteolysis, and recurrent fractures after minor trauma. The older sibling had the more severe skeletal phenotype, with 14 fractures involving both tibiae, the right femur, and right metatarsals. Most fractures were initially managed conservatively, but later injuries required plate-and-screw fixation because intramedullary nailing was not feasible due to marked bone sclerosis and near obliteration of the medullary canal. The younger sibling showed a milder course, with four tibial fractures treated orthopedically. Radiographs in both patients demonstrated diffuse osteosclerosis, persistent patency of cranial sutures, Wormian bones, mandibular hypoplasia, and distal acro-osteolysis, while calcium-phosphate parameters remained within normal limits. The diagnosis of pycnodysostosis was established on clinicoradiologic grounds. These sibling cases highlight the marked intrafamilial variability of pycnodysostosis and the technical difficulties of fracture management in sclerotic bone. Early recognition of the characteristic phenotype is essential to avoid misdiagnosis and to guide long-term multidisciplinary follow-up with individualized orthopedic planning.

## Introduction

Pycnodysostosis is a rare autosomal recessive skeletal dysplasia caused by defects in cathepsin K (CTSK) on chromosome 1q21. Although the bones are diffusely sclerotic, they remain brittle and prone to fracture [1]. The disorder is characterized by a distinctive but variably expressed phenotype that may include occipital bossing, micrognathia, an obtuse mandibular angle, exophthalmos, and a narrow palate [2]. From a diagnostic standpoint, the combination of osteosclerosis and acro-osteolysis of the distal phalanges is highly suggestive of pycnodysostosis and helps distinguish it from other forms of osteopetrosis, thereby helping avoid inappropriate management [3,4]. Clinically, the major orthopedic burden is related to recurrent low-trauma fractures, often with delayed healing, most commonly involving the femur and tibia [5]. Imaging may also demonstrate persistent open cranial sutures, diffuse femoral, tibial, and vertebral densification, and acro-osteolysis [2]. Management is multidisciplinary and depends on the manifestations; fracture treatment is frequently surgical, most commonly using plate fixation or intramedullary fixation, although intramedullary techniques may be technically difficult because of marked bony sclerosis [2]. Long-term follow-up is important because of the risk of refracture.

In this report, we present the cases of two sibling, born to consanguineous parents, admitted to our department for the management of fractures occurring after minor trauma.

## Case presentation

Case 1

**Figure 1:**
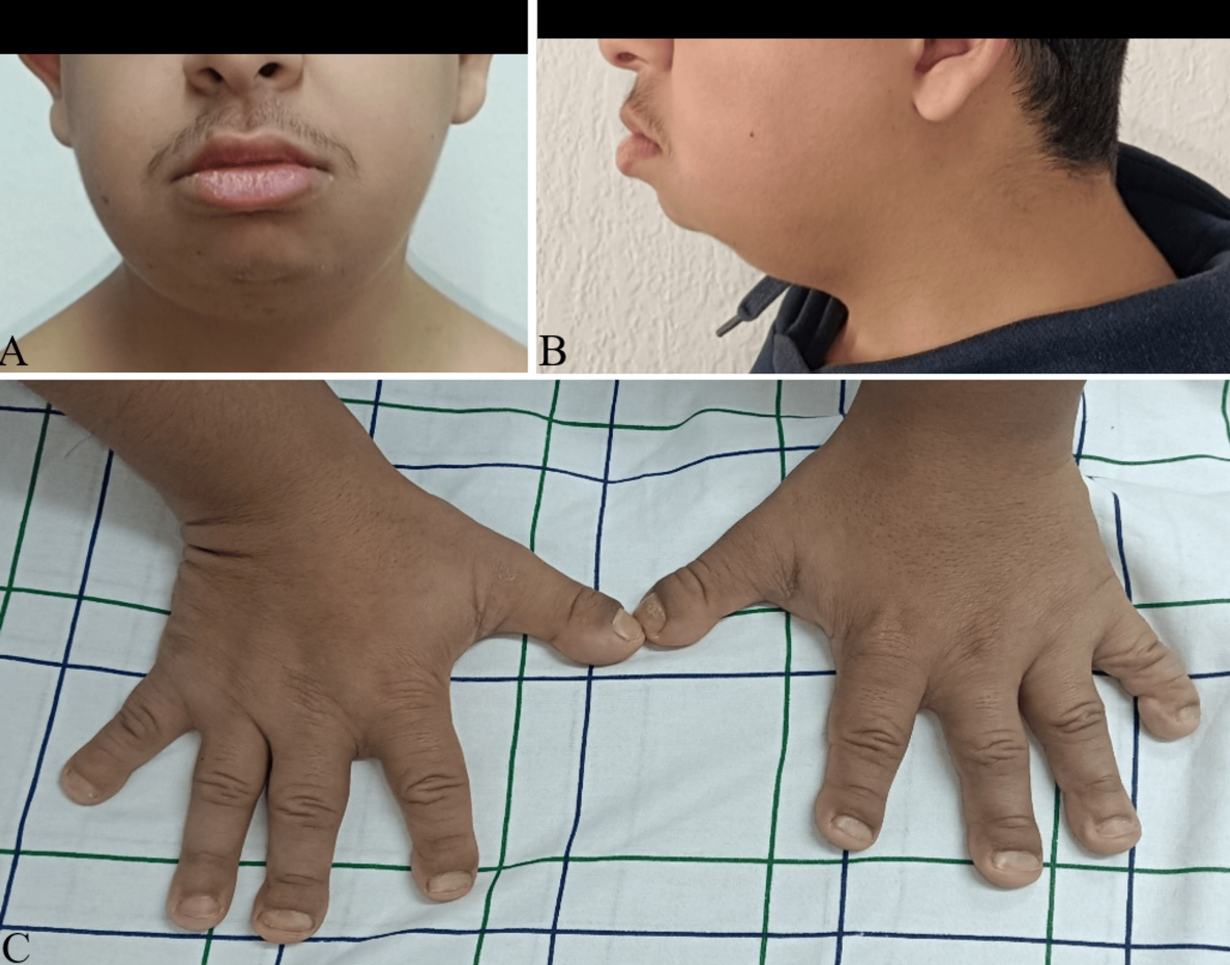
Clinical photographs showing facial features in frontal (A) and lateral (B) views, including frontal and occipital bossing, micrognathia, and a prominent nose. Broad, short hands with shortened fingers and curved nails are also shown (C).

Patient 1 was the older brother and the second of three siblings. He was 20 years old at the most recent evaluation. He had been followed since the age of four years for growth retardation and recurrent fractures. He had short stature, measuring 151.5 cm and weighing 45 kg. Clinical examination revealed a dysmorphic syndrome characterized by frontal and occipital bossing, persistent anterior fontanelle, exophthalmos, facial hypoplasia with microretrognathia, and a prominent nose. The hands and feet were broad and short, with shortening of the fingers and toes and curved nails (Figure 1).

**Figure 2:**
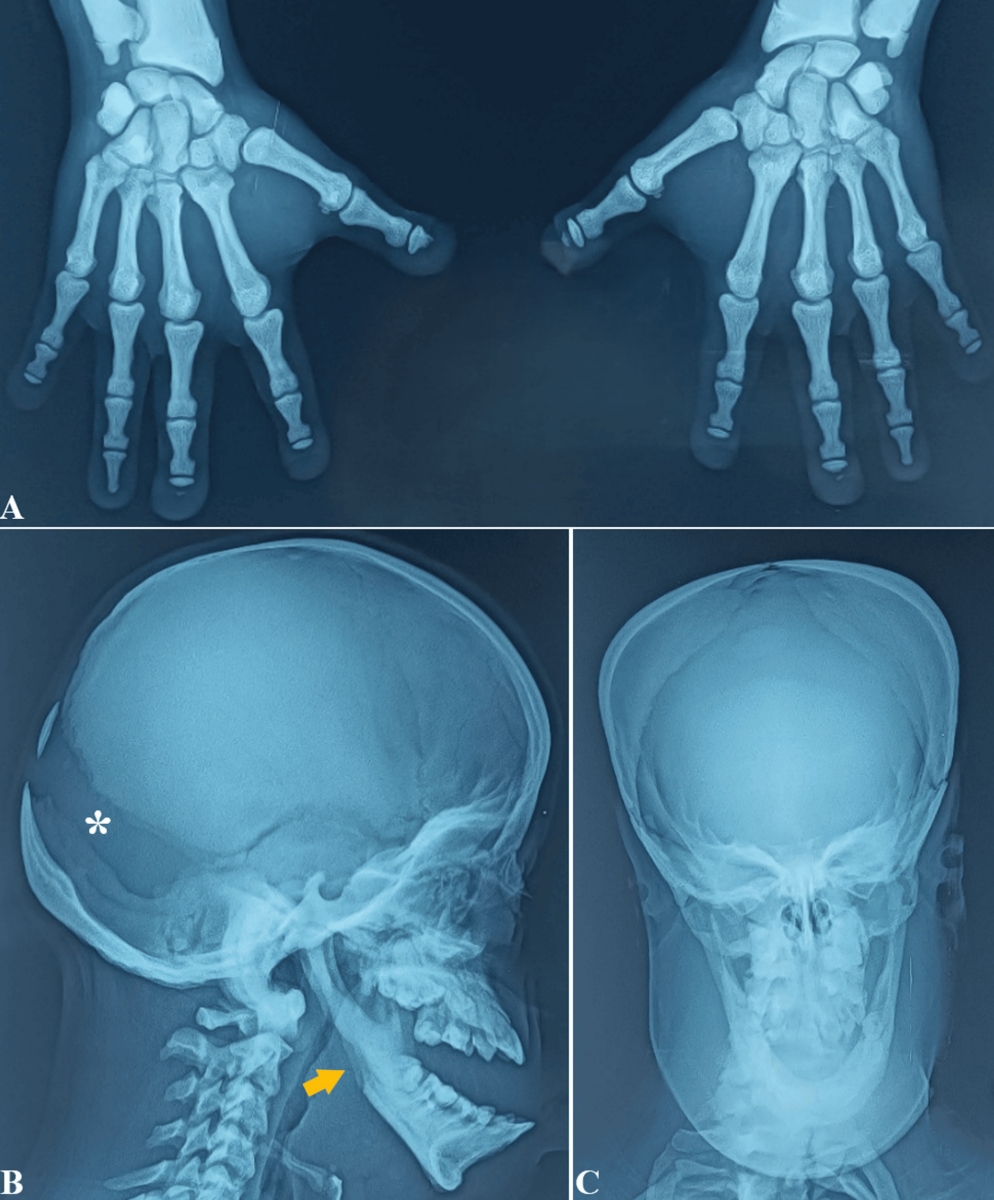
Hand radiograph showing acro-osteolysis of the distal phalanges, with sparing of the fourth digits (A). Skull radiographs show sclerosis of the skull base, widened cranial sutures, particularly the lambdoid suture (*), and loss of the mandibular angle (arrow) (B: lateral view; C: frontal view).

Radiographs showed diffuse osteosclerosis involving the skull base, persistent patency of the anterior fontanelle, widened cranial sutures, particularly the lambdoid suture, occipital Wormian bones, mandibular hypoplasia with loss of the mandibular angle, hypoplasia of the acromial end of the clavicle, and thoracic scoliosis. Hand radiographs showed shortened proximal and middle phalanges, with acro-osteolysis of the distal phalanges, sparing the fourth digits (Figure 2).

**Figure 3:**
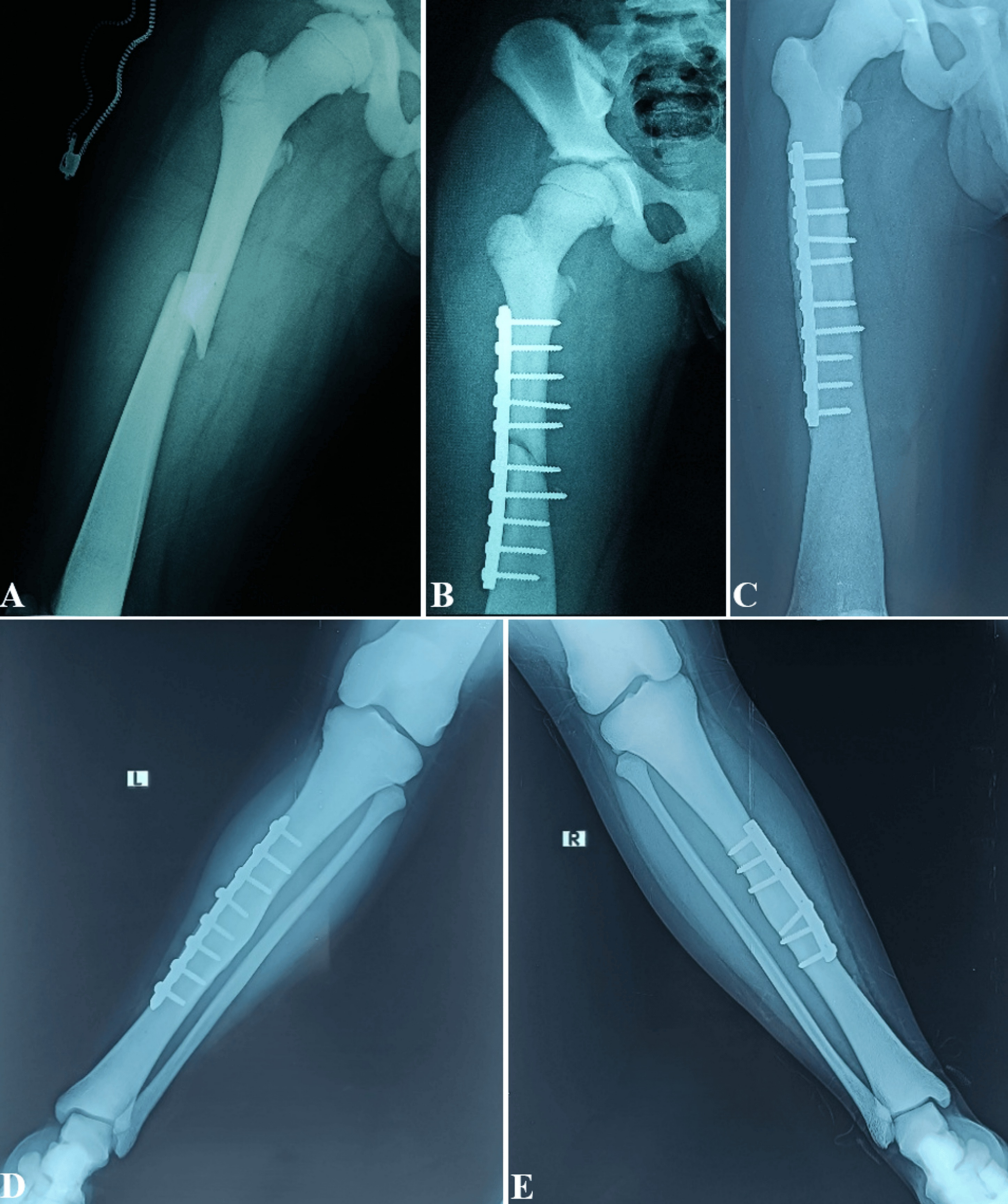
Lower-limb radiographs showing a right femoral fracture sustained at the age of 15 years (A), treated with plate fixation, with immediate postoperative imaging (B) and the six-year follow-up imaging (C). Follow-up radiographs obtained two years after surgery show left tibial (D) and right tibial (E) fractures sustained at the age of 18 years.

Long-bone radiographs demonstrated marked diaphyseal and metaphyseal sclerosis, predominantly in the lower limbs, associated with multiple healed fracture calluses. To date, the patient had sustained 14 fractures: six fractures of the right tibia, five fractures of the left tibia, two fractures of the right metatarsals, and one fracture of the right femur. The tibial and metatarsal fractures recurred repeatedly at the same sites. All fractures occurred after minimal trauma and were non-displaced, except for the femoral fracture. Most fractures were initially treated conservatively with prolonged cast immobilization, lasting up to nine months for the lower limbs. The most recent fractures were treated surgically to avoid prolonged immobilization. These included a displaced right femoral fracture at the age of 15 years and two simultaneous non-displaced fractures of both tibiae at the age of 18 years. Plate-and-screw fixation was performed because intramedullary nailing was not feasible due to marked bone sclerosis and a very narrow, nearly obliterated medullary canal (Figure 3).

Laboratory investigations showed a normal calcium-phosphate profile, with serum calcium at 96 mg/L, phosphorus at 50 mg/L, and alkaline phosphatase at 297 IU/L (Table 1).

**Table 1:** Laboratory assessment of calcium-phosphate metabolism in the two patients.

Parameter	Reference range	Patient 1	Interpretation	Patient 2	Interpretation
Serum calcium	85–106 mg/L	96 mg/L	Normal	92 mg/L	Normal
Serum phosphorus	40–70 mg/L	50 mg/L	Normal	48 mg/L	Normal
Alkaline phosphatase	85–400 IU/L	297 IU/L	Normal	245 IU/L	Normal

Case 2

**Figure 4:**
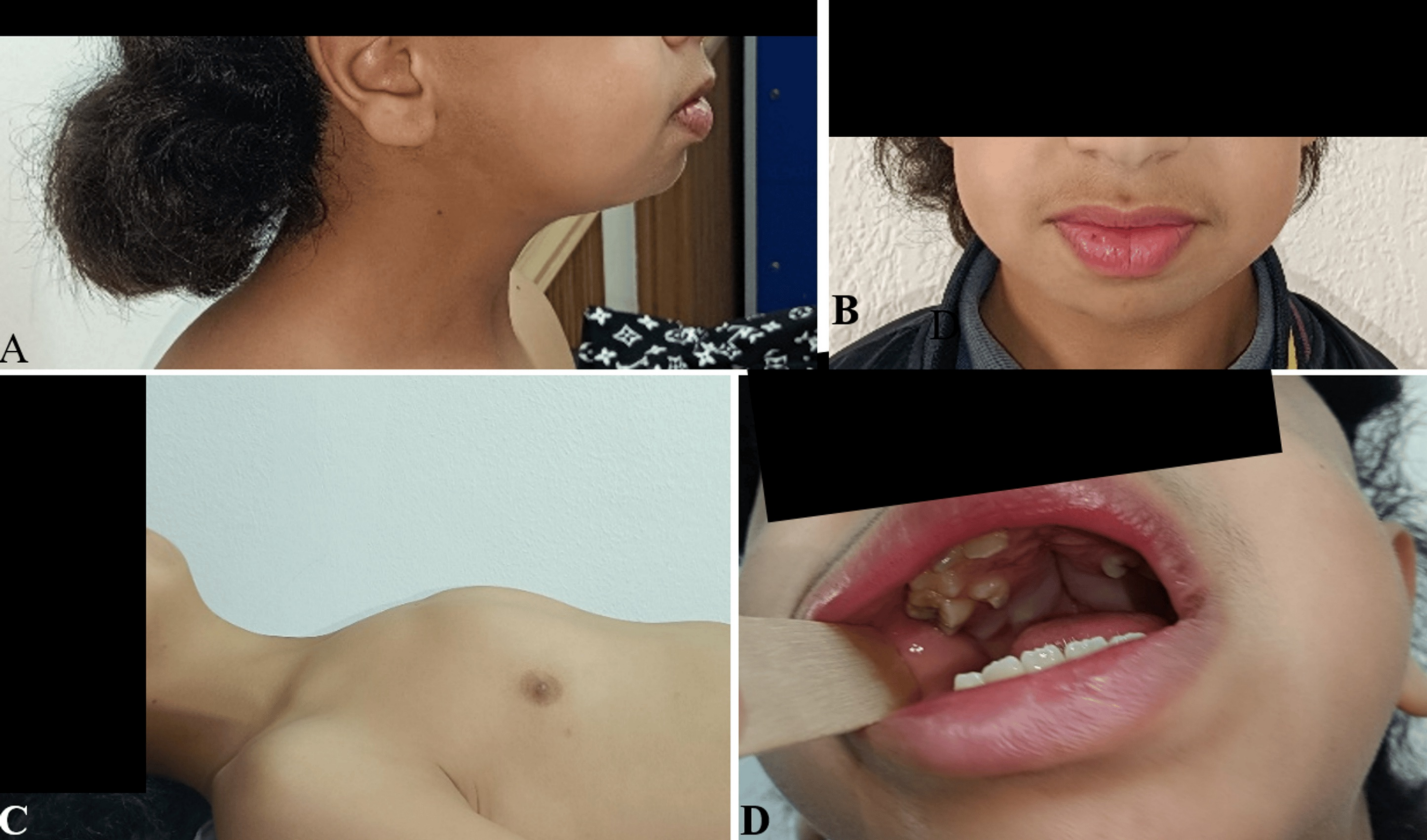
Clinical photographs showing facial features in lateral (A) and frontal (B) views, including frontal and occipital bossing, micrognathia, and a prominent nose. Pectus carinatum with microretrognathia (C) and narrow palate with dental malalignment and caries (D) are also seen.

Patient 2 was the younger brother and was 15 years old at the most recent evaluation. He had also been followed since childhood for short stature and recurrent fractures, but his skeletal course was milder than that of his older brother. Clinical examination revealed similar craniofacial dysmorphism, including frontal and occipital bossing, exophthalmos, facial hypoplasia with microretrognathia, and a prominent nose. He also had broad and short hands and feet, shortening of the fingers and toes, curved nails, a narrow palate, dental malalignment, and multiple dental caries. An asymmetric pectus carinatum was noted (Figure 4).

**Figure 5:**
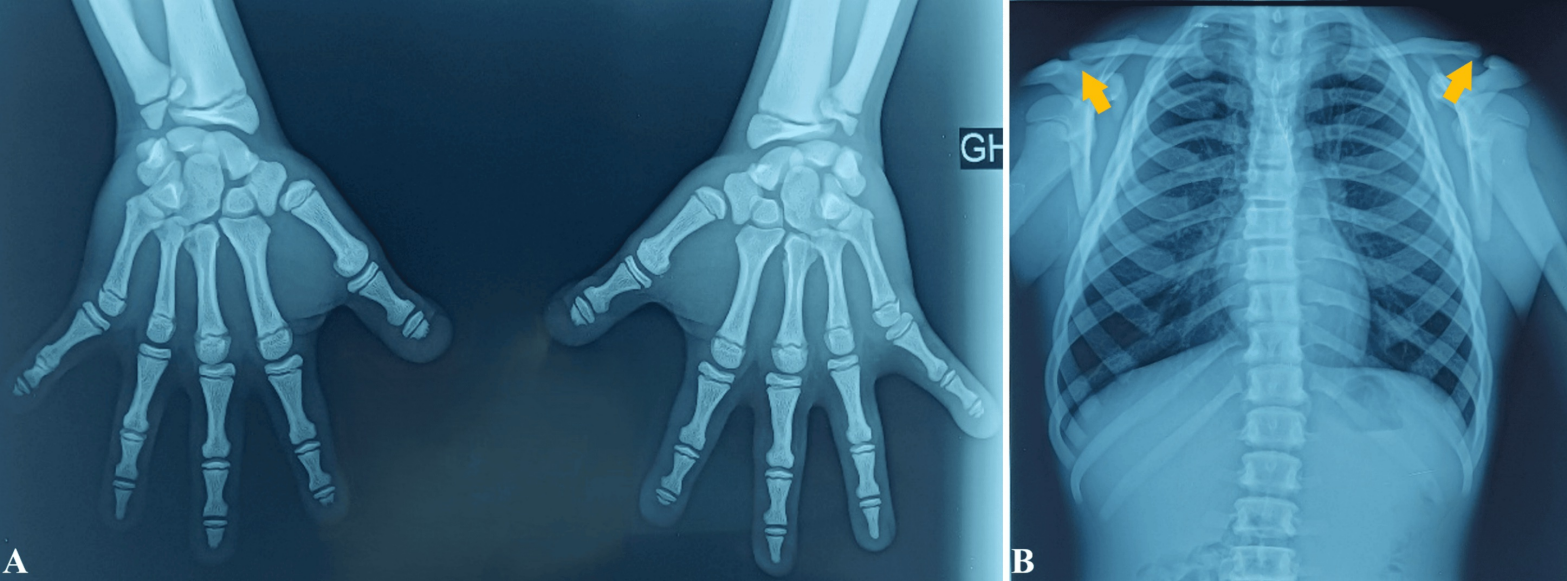
Hand radiograph showing acro-osteolysis of the distal phalanges, with sparing of the third and fourth fingers (A). Thoracic radiograph showing hypoplasia of the acromial ends of the clavicles (arrows) (B).

Radiographs showed diffuse osteosclerosis, persistent cranial suture widening, mandibular hypoplasia with loss of the mandibular angle, and hypoplasia of the acromial end of the clavicle. Hand radiographs showed shortening of the proximal and middle phalanges with acro-osteolysis of the distal phalanges, sparing the third and fourth fingers (Figure 5).

**Figure 6:**
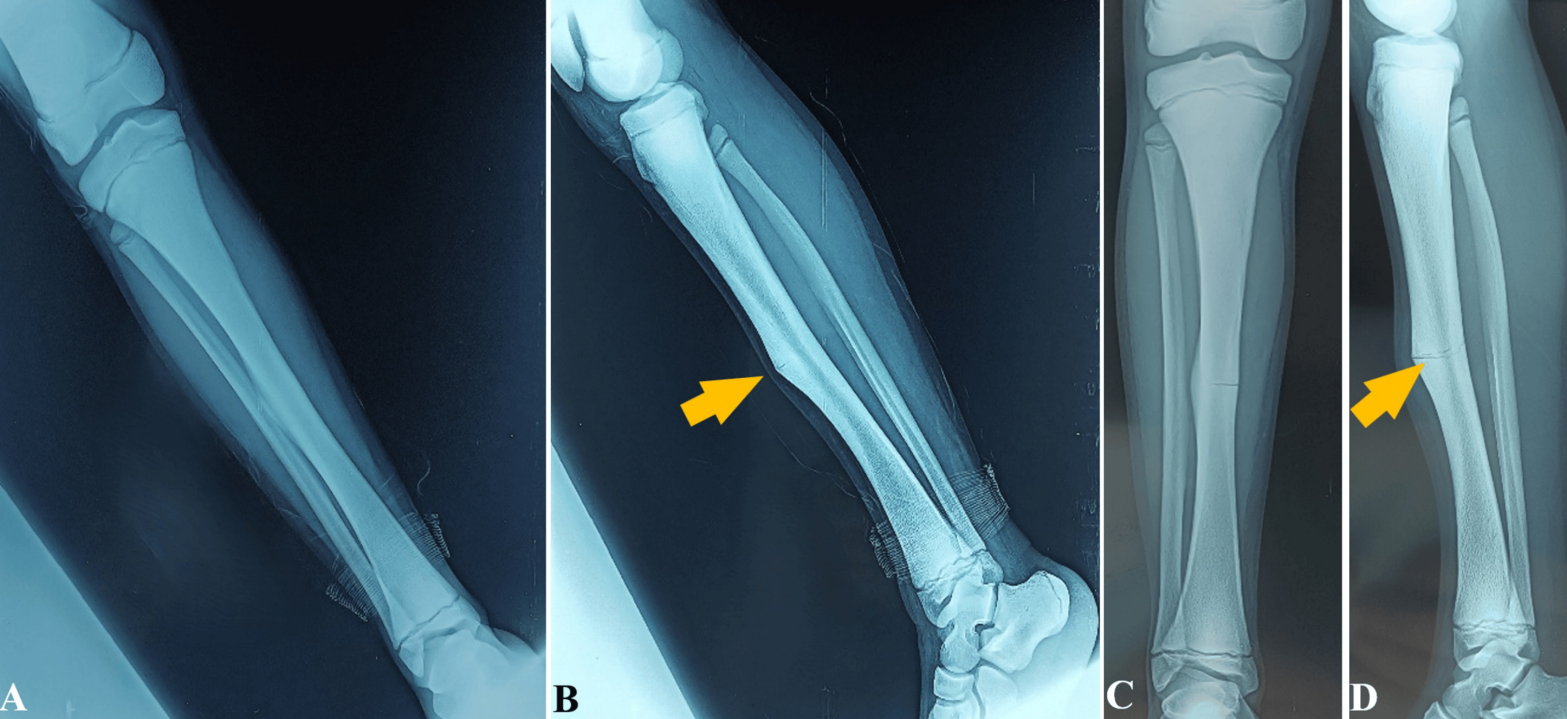
Lower-limb radiographs showing diaphyseal–metaphyseal sclerosis (A), a mid-diaphyseal tibial fissure with callus formation at the age of 15 years (arrow) (B), and a nondisplaced mid-diaphyseal fracture of the right tibia with prominent hypertrophic callus related to previous fractures at the same site (arrow) (C: frontal view; D: lateral view).

The patient began to sustain fractures at the age of seven years. He had experienced four fractures in total: one involving the left tibia and three involving the right tibia. All fractures occurred after minimal trauma and were treated orthopedically with prolonged cast immobilization. Lower-limb radiographs showed diaphyseal and metaphyseal sclerosis, a mid-diaphyseal tibial fissure with callus formation, and a non-displaced mid-diaphyseal fracture of the right tibia with prominent hypertrophic callus related to previous fractures at the same site (Figure 6).

Laboratory investigations showed a normal calcium-phosphate profile, with serum calcium at 92 mg/L, phosphorus at 48 mg/L, and alkaline phosphatase at 245 IU/L (Table 1).

The diagnosis of pycnodysostosis was established based on the characteristic clinical and radiological findings. Unlike his older brother, he had so far been managed exclusively with orthopedic treatment and regular follow-up. Genetic counseling and dental care were proposed to the family.

## Discussion

Pycnodysostosis is a rare autosomal recessive skeletal dysplasia with an estimated incidence of approximately one per million. Its frequency is increased in consanguineous populations, with no clear sex predominance [2,5]. The disorder results from mutations affecting cathepsin K (CTSK), located on chromosome 1q21, which is expressed in osteoclasts and plays a key role in the degradation of bone matrix proteins. Deficiency of this enzyme leads to abnormal bone remodeling, osteosclerosis, and bone fragility [2]. Because facial dysmorphism may be subtle, especially in younger patients, pycnodysostosis may initially be underrecognized or misdiagnosed [4].

The clinical phenotype is variable but classically includes short stature, increased bone fragility, craniofacial dysmorphism, and acro-osteolysis. Facial features such as mandibular hypoplasia, aquiline nose, micrognathia, occipital bossing, obtuse mandibular angle, exophthalmos, and narrow palate are frequently reported and may suggest the diagnosis early in life [6]. Maxillofacial abnormalities are also prominent manifestations of the disease [7]. Fractures often occur after minimal trauma or spontaneously, most commonly involving the femur and tibia, and may show delayed healing [1]. In addition, osteosclerosis, shortened distal phalanges, and loss of the mandibular angle are characteristic findings [5]. Craniosynostosis may occur and, in some patients, may lead to optic atrophy and visual impairment [8]. Conversely, the absence of acro-osteolysis can lead to confusion with osteopetrosis [9]. Upper airway manifestations such as stridor and laryngomalacia have also been described and may contribute to earlier clinical suspicion [10].

Radiologically, pycnodysostosis is characterized by generalized bone sclerosis, vertebral notching, and acro-osteolysis of the distal phalanges [2]. The combination of increased bone density and acro-osteolysis is considered highly suggestive, if not almost pathognomonic, of the disease. Other useful imaging clues include delayed closure of cranial sutures and non-pneumatized mastoids [10]. Brain MRI may be useful in selected patients to screen for associated Chiari malformation [8,10]. In clinically suggestive cases, molecular confirmation by CTSK testing remains valuable [4].

Fracture management in pycnodysostosis remains challenging. Both conservative and surgical strategies have been reported, although treatment is often complex and surgery-heavy [10]. Surgical options include plate fixation, intramedullary fixation, and Ilizarov external fixation [2]. However, operative management is technically demanding because of the dense sclerotic bone, thick cortex, and narrow medullary canal, which may complicate drilling and insertion of intramedullary devices [11]. In addition, the risk of difficult intubation should be considered before anesthesia [10].

Published orthopedic data highlight these challenges. A systematic review by Taka et al. reported that most fractures in pycnodysostosis were treated surgically, with plate fixation being the most common technique, whereas intramedullary fixation was associated with the lowest refracture rate in the reviewed cases [2]. Bor et al. favored intramedullary nailing when feasible and suggested retaining the hardware after union to reduce the risk of future refracture [12]. Rovira Martí et al. reported nonunion in two of five patients and emphasized that intramedullary nailing may be difficult or impossible because of skeletal sclerosis, requiring alternative strategies [13]. Similarly, Sharma et al. described technical difficulty with titanium elastic nailing in a pediatric femoral fracture because of severe metaphyseal sclerosis, ultimately requiring plate fixation, and recommended anticipating the need for open fixation and multiple drill bits during surgery [14].

Management should also address associated non-orthopedic complications. Sleep apnea, dental abnormalities, and oral complications are common and may require multidisciplinary care, including non-invasive ventilation when indicated [10]. Hearing loss has also been reported [10]. In selected patients with growth hormone deficiency, growth hormone therapy has been proposed to improve linear growth [10], particularly since nearly half of affected individuals may show this deficiency [4].

Overall, although pycnodysostosis is not usually life-threatening, it may be associated with substantial morbidity related to recurrent fractures, delayed union, malunion, postoperative complications, craniofacial abnormalities, respiratory problems, and dental disease [4,5]. Therefore, patients benefit from long-term multidisciplinary follow-up, involving orthopedics, neurosurgery, orthodontics, respiratory medicine, and rehabilitation, in order to optimize functional outcomes and reduce complications [4].

## Conclusions

Pycnodysostosis is a rare skeletal dysplasia that should be suspected in patients presenting with recurrent low-trauma fractures, short stature, osteosclerosis, acro-osteolysis, and characteristic craniofacial dysmorphism. These two sibling cases illustrate the marked clinical variability of the disorder, even within the same family, particularly in terms of fracture burden and orthopedic course. They also highlight the practical challenges of fracture management in dense, brittle bone, where intramedullary fixation may be technically impossible because of severe medullary canal narrowing. Early recognition of the clinicoradiologic features is essential to avoid misdiagnosis and inappropriate treatment, while long-term multidisciplinary follow-up remains crucial to optimize orthopedic outcomes and address associated craniofacial, dental, and functional complications.
